# Dancing With Health: Quality of Life and Physical Improvements From an EU Collaborative Dance Programme With Women Following Breast Cancer Treatment

**DOI:** 10.3389/fpsyg.2021.635578

**Published:** 2021-02-24

**Authors:** Vicky Karkou, Irene Dudley-Swarbrick, Jennifer Starkey, Ailsa Parsons, Supritha Aithal, Joanna Omylinska-Thurston, Helena M. Verkooijen, Rosalie van den Boogaard, Yoanna Dochevska, Stefka Djobova, Ivaylo Zdravkov, Ivelina Dimitrova, Aldona Moceviciene, Adriana Bonifacino, Alexis Matua Asumi, Dolores Forgione, Andrea Ferrari, Elisa Grazioli, Claudia Cerulli, Eliana Tranchita, Massimo Sacchetti, Attilio Parisi

**Affiliations:** ^1^Research Centre for Arts and Wellbeing, Edge Hill University, Ormskirk, United Kingdom; ^2^University Medical Centre Utrecht, Utrecht, Netherlands; ^3^Bulgarian Sports Development Association, Sofia, Bulgaria; ^4^Klaipeda Regional Women's Information Center, Klaipeda, Lithuania; ^5^IncontraDonna, Rome, Italy; ^6^Istituto Europeo per lo Sviluppo Socio Economico, Alessandria, Italy; ^7^Department of Human Movement Sciences and Health, Università degli Studi di Roma Foro Italico, Rome, Italy

**Keywords:** physical activity, dance, dance movement therapy, breast cancer, evaluation

## Abstract

**Background:** Women's health has received renewed attention in the last few years including health rehabilitation options for women affected by breast cancer. Dancing has often been regarded as one attractive option for supporting women's well-being and health, but research with women recovering from breast cancer is still in its infancy. Dancing with Health is multi-site pilot study that aimed to evaluate a dance programme for women in recovery from breast cancer across five European countries.

**Methods:** A standardized 32 h dance protocol introduced a range of Latin American dances presented within a sports and exercise framework with influences from dance movement therapy. Fifty-four women (M age 53.51; SD 7.99) participated in the study who had a breast cancer diagnosis <3 years, chemotherapy >6 weeks, no indication of metastasis, or scheduled surgery/chemotherapy/radiation treatment for the duration of the intervention. Primary outcome data was collected for anthropometric and fitness measures next to cancer-related quality of life. *T*-tests and Wilcoxon signed ranked tests were used to establish differences pre and post intervention. Cohen's d was also calculated to determine the effect size of the intervention.

**Results:** Statistically significant changes were found for: (i) weight, right and left forearm circumference and hip; (ii) 6 min walking, right and left handgrip, sit-to-stand and sit-and-reach; (iii) the EORTC-QLQ C30 summary score as well as the subscales of emotional and social functioning and symptoms. In all cases the direction of change was positive, while Cohen's d calculated showed that the effect of the intervention for these parameters ranged from intermediate to large.

**Conclusion:** Changes on the above anthropometric, fitness and quality of life measures suggest that the intervention was of value to the participating women recovering from breast cancer. Results also advocate collaborative efforts across countries to further research.

## Introduction

### Breast Cancer

Breast cancer impacts over two million women each year with global rates increasing rapidly (Global Cancer Observatory, 2018). It is the most prevalent type of cancer in women, with women in developed countries having the highest incidences. In 2018 for example, more than 560,000 women were affected by breast cancer in Europe alone, with rates varying from five to 10 percent depending on the country (Ferlay et al., 2018). The World Health Organisation Europe WHO/Europe (2020) argues that in the period between 1950 and late 1980s, there was a considerable increase in mortality due to breast cancer in most European countries with a peak in rates in the 1990s. Since then, for some countries' mortality rates have remained stable (e.g., Lithuania and Bulgaria), whilst for others there have been decreased (e.g., UK, the Netherlands, and Italy) (Autier et al., 2010). These changes on mortality rates may have been the result of public measurements (e.g., self-exams and screening mammograms performed annually after women reach their 40's) that have been promoted in the last decades to detect an early development of breast cancer.

Risk of developing breast cancer is strongly associated with a range of factors including age, family history, reproductive and gynecological history, and lifestyle (Sun et al., 2017). Women over 40 with a family history of breast cancer have a substantial risk burden of developing the disease themselves (Brewer et al., 2017; Siegel et al., 2017). In addition, a history of early menarche, late menopause or prima gravida (giving birth late for the first time) also increases a woman's risk of breast cancer. Further specific risk factors include pre-menopausal estrogen levels (Dall and Britt, 2017; Horn and Vatten, 2017), oral contraceptive use (Bethea et al., 2015; Soroush et al., 2016), and using hormone replacement therapy (Beral and Million Women Study Collaborators, 2003; Narod, 2011; Liu et al., 2016). Finally, modern lifestyle impacts for increasing breast cancer risk include: excessive alcohol use (Hamajima et al., 2002; Jung et al., 2016), a diet high in saturated fats (Makarem et al., 2013), and smoking (Catsburg et al., 2015; Gaudet et al., 2017; Kispert and McHowat, 2017; Knight et al., 2017). A clear link between physical activity and risk for breast cancer is also reported in the literature (Friedenreich and Cust, 2008) with high levels of physical activity being linked with a decreased risk. This constellation of physiological, social, and genetic risk are often seen as intricately linked with poor overall prognosis.

Breast cancer treatment involves stratified treatments, which may include surgery (including breast conserving therapy, mastectomy, sentinel-node biopsy, and axillary dissection), radiotherapy, chemotherapy with or without anti-HER2 therapy (i.e., a blocker of the function of the protein in tumors whose HER2 gene is stuck on overdrive) and endocrine therapy (Senkus et al., 2015; National Institute for Health and Care Excellency (NICE), 2018), all of which have long-term impacts on women's health and well-being. For many women, the experience from diagnosis to treatment involves not only acceding to such medical and surgical procedures with their physical consequences, but also experiencing psychological and mental health sequalae. Physically, women may experience lymphoedema (swelling in the breast, arm or hand that can feel tight, heavy or full), which can be painful (Johansen et al., 2014) or increase the risk of serious infections (Fu, 2014). Shoulder problems (Stubblefield and Keole, 2014), fatigue (Berger et al., 2012), and redness of skin (Ryan et al., 2013) are also common physical symptoms; and there is further increased risk of cardiac and respiratory problems amongst some cancer survivors (Darby et al., 2005). Chemotherapy and endocrine therapy, although often a key response to treating breast cancer, can prove a serious insult leading to polyneuropathy (Grisold et al., 2011), early menopause, hot flushes and mood swings (Col et al., 2005), osteoporosis (Hamood et al., 2019), and weight gain (Vance et al., 2011). Cumulatively, the impact of these surgical and medical treatments can lead, not surprisingly, to a deterioration in quality of life for many women (Marino et al., 2008).

It has been argued that quality of life can be affected negatively from the time of initial diagnosis and treatment (Mols et al., 2005) to long after treatment has ended (Burgess et al., 2005). Cognitive function can decrease leading to what is called “chemo-fog,” affecting memory and concentration (Janelsins et al., 2017). Persisting fatigue and asthenia during radiotherapy and chemotherapy can serve to amplify existential anxiety and distress, thus decreasing the patient's quality of life (Schneider et al., 2003) and increasing symptoms of anxiety and depression. Overall, women with breast cancer report more symptoms of anxiety and depression, especially if they have gone through chemotherapy (Lim et al., 2011), with depression and anxiety persisting for a large proportion of women even 5 years after diagnosis and treatment (Burgess et al., 2005). Indeed, more recent studies suggest even longer term effects on mental health, highlighting that as treatment and survival rates increase, so will rates of long-term depression and anxiety in cancer survivors (Niedzwiedz et al., 2019).

Given the above issues, it is important that sufficient attention is given to offering appropriate support that addresses both the psychosocial and the physical concerns experienced by women surviving breast cancer. Physical activity including dance can potentially address some of the physical concerns raised, and when this is done sensitively, relevant literature suggests that dance may also have a positive impact on psychosocial outcomes.

### Physical Activity

Recent studies suggest that physical activity can have positive effects on cancer prevention, with leisure-time physical activity being associated with lower risk for many cancer types; and in the main this is regardless of body size or smoking history (Moore et al., 2016). More specifically, for breast cancer, being physically active has positive benefits for both prevention and treatment (Graf and Wessely, 2010), with physical activity and weight loss closely linked with reducing the risk for breast cancer (Hardefeldt et al., 2018). Indeed, increased physical activity can decrease the risk for breast cancer by 25–30% on average, while recreational activity, lifetime engagement in physical activities and vigorous activity are regarded as important for prevention (Friedenreich and Cust, 2008).

Furthermore, Lahart et al. (2015) suggest that there is lower risk of developing breast cancer not only amongst those who engage with physical activity as a lifetime recreational activity, but physical activity is also highly beneficial post diagnosis. Grazioli et al. (2017) argue that physical activity positively affects the course of breast cancer, reducing the risk of recurrences possibly due in some part to exercise's mediating effects on hormonal and genetic expressions. The recommended physical activity guidelines by WHO (World Health Organisation) (2020) of 150 min per week of moderate intensity aerobic physical activity or equivalent are often cited as an important dosage post diagnosis, possibly due to the well-reported benefits of exercise as a result of increased endorphin production: improved cardiorespiratory fitness, musculoskeletal strength, and improvements in well-being and quality of life (Rock et al., 2020). Specific research in the dosage relevant for women recovering from breast cancer also draws attention to the volume and intensity of exercise that is relevant to the different stages of recovery from the breast cancer, mainly because of impairments of the immune system from the disease and associated treatment and the immune responses from exercise training. Lopez et al. (2020) meta-regression analysis for example, suggests that low volume resistance training may be superior to high volume resistance training for increasing muscle strength amongst breast cancer patients undergoing primary treatment.

Although physical activity can have a significant impact both mentally and physically (Cancer Research UK, 2020), few women surviving breast cancer regularly engage in physical activity (Rethorst et al., 2018). This could be the result of cancer-related fatigue. For women with breast cancer, sleep deficiency is common and distressing throughout their care. Physical and body-mind activities are promising responses for addressing sleep problems amongst breast cancer patients (Kreutz et al., 2019). However, further studies are needed to clarify type and dosage. Amongst the different types of physical activity, dance is an attractive and potentially acceptable form of physical engagement amongst women survivors of breast cancer as the following section shows.

### Dance

As a physical activity, dance is often regarded as a fun and engaging activity that can arouse spontaneity, eliminate tension and increase body awareness (Malicka et al., 2011). Physically, dancing can release endorphins (Lovatt, 2020), and it can have positive effects on pain threshold (Tarr et al., 2015) which could support pain management. Psychologically it can boost self-esteem, and act as an outlet for pent up emotions (Jola and Calmeiro, 2017). As a social activity it provides opportunities for social connections and support and can be seen as an effective group bonding activity (Tarr et al., 2015). Inherent to dance is the presence of music, which has been reported to have direct impact on physical outcomes (Terry et al., 2020).

Furthermore, dance can address cancer-related fatigue and improve quality of life (Sturm et al., 2014). In a study of women with breast cancer completed by Malicka et al. (2011) it was highlighted that from a range of different activities on offer, dance alongside trips, were the most important activities for improving quality of life. If dance activities are well-structured, in terms of intensity, duration, and frequency, evidence shows that dance improves physical and psychosocial outcomes amongst breast cancer patients. Boing et al. (2017) in their systematic review of studies on dance for breast cancer, have argued that ballroom (Pisu et al., 2017), Greek folk (Kaltsatou et al., 2011), ballet and jazz (Molinaro et al., 1986), mindful movement (Crane-Okada et al., 2012), and sacred dance (Frison et al., 2014) have all led to both physical (increased range of motion and strength in upper limb and functional capacity) as well as psychosocial improvements (quality of life, self-image, femininity, mood, self-esteem, consciousness, and perceived physical well-being). They can also contribute to the reduction of psychological concerns (stress, pain, depression, anxiety and fear). Their conclusion was that dance can be an effective alternative adjuvant treatment in breast cancer.

Boing et al. (2017) study also summarizes the duration and frequency of the dance programmes in the reviewed studies. These include 3 to 24 weeks, one to three times per week with sessions lasting from 1 to 3 h each. They also suggest that for participants to receive the best physical benefits from the dance classes, they need to be involved in either 1 h long sessions three times a week or a 3 h session once a week. Moderate intensity is also recommended as falling within relevant guidelines and in accordance with WHO (World Health Organisation) (2020) recommendations referred to in the previous section.

Amongst the studies reviewed by Boing et al. (2017), it is worth highlighting the study by Pisu et al. (2017) because of the form of dance used and thus, its relevance to this study. Thirty-one breast cancer patients and their partners took part in a ballroom dance group intervention that included foxtrot, waltz, cha-cha-cha and east coast swing. It was noted that participants enjoyed spending time to move together and saw this activity as supporting them to become more physically active, improve functional capacity and quality of life. In addition to the dimensions of physical and emotional well-being, trust between the couples was built, highlighting the effect of the dyadic and social components of dance as important outcomes in this study.

More recent studies highlight similar outcomes: a small pilot study by Loo et al. (2019) demonstrated that participation is a cultural dance programme led to sustainable increases in levels of physical activity in cancer survivors as well as improvements in quality of life and vigor. A further benefit was decreased cytokine levels: important markers for inflammatory processes, pathological pain, and chronic inflammatory states associated with obesity. In another dance study, breast cancer patients attended a 12 week belly dance programme offering a viable physical activity, which was reported as offering associated benefits for quality of life, levels of fatigue, and depressive symptoms (Boing et al., 2018). Whilst in this case there was not a significant difference between the experimental and control group, the findings were sufficient to support a further larger trial that awaits published results.

It appears then that studies focusing on dance for women with breast cancer are growing in number and offering promising results. So too are studies in the more specialized discipline of dance movement therapy, which is described further in the following section.

### Dance Movement Therapy

Dance movement therapy is a form of psychotherapy offering “individuals of all ages and abilities a space to explore what drives them, assisting people to develop self-awareness and sensitivity to others and also to find a pathway to feeling more comfortable in their own skin” (European Association for Dance Movement Psychotherapy, 2020, p. 1). Based on the premise that body and mind are connected and that changes in the one informs changes in the other (Karkou and Sanderson, 2006), it requires specific training, and has a growing evidence base. Effectiveness studies suggest that dance movement therapy can be useful for diverse populations, and has significant benefit for those with depression (Meekums et al., 2015; Karkou et al., 2019) and also in anxiety (Bräuninger, 2012).

Further support for using dance movement psychotherapy in breast cancer care is found in the Cochrane Review of Bradt et al. (2015), within which three randomized controlled trials from Dibbell-Hope (1989), Sandel et al. (2005), and Ho et al. (2016) evidence that dance movement therapy is well-tolerated, with small dropout rates. This Cochrane Review called for further studies to strengthen claims of dance movement psychotherapy's effectiveness for depression, anxiety, fatigue, and on improving body image (poor body image being a common issue after mastectomy). Results from Sandel et al. study 2005 suggested (with moderate risk of bias) that dance movement therapy had a large beneficial effect on the quality of life of 37 participants, while improvement in vigor and reduction of somatization was reported by Dibbell-Hope (1989; *N* = 31).

A later systematic review by Boing et al. (2017) considered a wider range of dance movement therapy studies alongside studies on dance. In addition, it reported on qualitative data—participants' experiences—as well as quantitative outcomes. One study in the review (Serlin et al., 2000) for example, highlighted significant patient perceived benefits, and improvements in several psychosocial aspects of breast cancer care. Another study (Ho et al., 2016) reporting qualitative results from a twelve-session intervention in China, found that dance movement therapy showed “significant effects on buffering the deterioration in perceived stress, pain severity, and pain interference” (p. 824). Similarly, qualitative results by Dibbell-Hope (2000) reported increased body awareness associated with acceptance of body and self, improvement in mood, and decrease of worry about the future. In addition, participants reported an overall sense of strength and ease within the context of social support. Further observations were that age and past experience with dance and sports, appeared to have an impact on satisfaction of body-image and self-esteem, supporting the view shared in studies on physical activity (Santa Mina et al., 2012) that, subject to medical clearance, the earlier women start being physically active or return to exercise (here dance) after breast cancer treatment the better.

After breast cancer surgery, be that mastectomy, lumpectomy, or mastectomy with breast reconstruction, or other medical interventions, many women experience anxiety, depression, and emotional distress. These issues can persist for many years after surgery, and often relate to changing body image, functional limitations, and weight gain; all of which can negatively affect their quality of life. Therefore, the importance of being physically active and receiving appropriate psychological support for women diagnosed with breast cancer cannot be overlooked. Research suggests it should be integrated as part of a holistic approach to recovery. However, despite a large research base evidencing positive effects for physical activity not only in prevention, but most importantly treatment (Blanchard et al., 2008; Mason et al., 2013) in breast cancer survivors, the majority of women diagnosed with breast cancer remain inactive.

Enjoyable and sociable physical activities such as dance, as well as creative arts therapies such as dance movement therapy that provide both physical and psychosocial benefits, are becoming increasingly relevant in supporting cancer patients with their recovery. The need to investigate this type of interventions further and thus offer additional and attractive options to women in their recovery have been reasons for conducting the current study.

### Research Questions and Overall Aim

The project asked the following questions:

– What is the impact of the therapeutic dance programme titled Dancing with Health on the physical health of women recovering from breast cancer with respect to anthropometric measures, cardiorespiratory fitness, muscle strength, balance, flexibility, and activity levels?
– What is the impact of the Dancing with Health programme on the psychosocial health of the women with respect to cancer-related quality of life?

Overall, the study aimed to evaluate the impact of the Dancing with Health programme on the physical functioning and psychosocial well-being of women in recovery from breast cancer. As a multi-site pilot study in five different countries, it was also intended to evaluate the feasibility of such a programme to support future research developments.

## Materials and Methods

### Study Design

This pilot study evaluated a standardized dance programme delivered in five countries, namely UK, Italy, Lithuania, Bulgaria, and the Netherlands. It followed a quasi-experimental design with pre and post intervention quantitative measures. Additional quantitative measures relating to body image, anxiety and depression, qualitative and arts-based data were collected from the UK cohort but these findings are not presented here.

The study was approved by the University Medical Centre Utrecht, Netherlands, and by the Research Ethics Committee of the Faculty of Health and Social Care at Edge Hill University, UK.

### Participants

Seventy women aged 30–65 years were recruited in total across the five participating European countries, with between 10 and 18 women recruited from each country. Participants were recruited from a range of settings including a University Medical Centre (Netherlands), non-profit women's organizations (Lithuania and Italy), a sports development institution (Bulgaria) and a university (UK). They were recruited through purposive sampling process using inclusion and exclusion criteria. Inclusion criteria involved: breast cancer diagnosis of fewer than 3 years, willingness and physical ability to take part in moderate physical activity, chemotherapy concluded at least 6 weeks before enrolling. Exclusion criteria involved: no indication of metastasis or scheduled surgery/chemotherapy/radiation treatment for the duration of the intervention.

### Intervention

The intervention involved delivering a standardized dance programme within a strong sports and exercise framework, informed by dance movement therapy principles. It combined an introduction to a range of dance styles (merengue, bachata, cha-cha, salsa, rumba, and tango) with exercise components. The content was developed by Università degli Studi di Roma Foro Italico in collaboration with Carolyn Smith, an international dance champion, teacher and television dance show judge (Italian Dancing with the Stars) who herself was treated for breast cancer. From a sports perspective it was considered on average to be a moderate intensity physical activity (i.e., causing participants to feel increased heart rate, respiration and body temperature, yet still able to hold a conversation (Woltmann et al., 2015) with the added value of being safe, psychologically minded, enjoyable, and social, to encourage a broad participation and a minimum drop-out. Influences from dance movement therapy offered psychological underpinnings to the work. In particular, the concept of safety, physical and psychological, was highlighted (European Association for Dance Movement Psychotherapy, 2020), the use of specific dances as metaphors for one's concerns and for life (Meekums, 2002), and opportunities to explore material creatively, while reflecting on the psychological meaning of these explorations (Karkou and Sanderson, 2006) shaped the manual of the intervention. The facilitators in each country were invited to engage with these principles depending on their background and qualifications. Also, carrying a client-centered ethos, to respond to the needs of the particular group of participants through adaptation. On the whole, the intervention became a dance practice with a strong therapeutic character (see other such examples in Karkou and Sanderson, 2006; Karkou et al., 2017 for differences between dance movement therapy and therapeutic dance).

The programme included 2 h/week for 16 weeks, and 32 h in total over a 4 month period between 2018 and 2020. Make-up sessions were given when necessary to account for inclement weather conditions or illness.

The sessions were organized as follows:

- Warm-up (10 min)
- Learning and performing dance routine (40 min)
- Cool-down (10 min)

During the main activity (40 min dance routine), although exercise intensity was not formally assessed, participants were encouraged to work at a cardio-respiratory intensity that they found challenging yet enjoyable and still able to respond verbally, corresponding to a moderate intensity of physical exertion (Woltmann et al., 2015). As diverse groups of individuals who experienced varying physical impacts of their cancer and subsequent treatment, it was necessary for the programme to respond to these individual needs, in line with safety principles (European Association for Dance Movement Psychotherapy, 2020). Therefore, rest and water breaks could be taken when desired and simplified or lower impact movements were offered as alternatives for those who found parts of the choreography too challenging or unsafe (for example turns). Facilitators used their best clinical judgement in dialogue with the participants to detect when groups required a water break. Choreography for each of the six dances in general was simple enough to be learned in one session, leaving ample additional time to build upon the basic choreography by increasing expressiveness, varying or increasing arm movements, interspersing travel around the room, using music with increased BPM and/or energy dynamics and generally increasing the more performative nature of the dance. According to Rodrigues-Krause et al. (2018), this “show” stage of dance corresponds to a more intense level of exertion (ventilatory threshold one), thus supporting and encouraging increases in activity intensity.

Over the duration of the delivering, the programme was organized in five stages that enabled the development of the group cohesion gradually influenced by the manualized intervention proposed in the Arts for the Blues project (Omylinska-Thurston et al., 2020). Latin American dance material was integrated in these five stages, informing the content of each session (mainly the middle part) as follows:

Stage 1: Focus on proprioceptive exercises and the perception of one's body in space. Breathing exercises to take diaphragmatic breathing consciousness from supine or sitting position. Reactivate joint mobility, in particular the shoulder. Individual work with the mirror, barefoot, and using a chair. All exercises were performed with a musical base, sometimes with eyes closed aiming to connect with one's self.
Stage 2: Rhythmic exercises to enhance body activation and promote coordination using the basic steps of merengue and bachata. It stimulated cognitive functions and encouraged participants to concentrate and memorize the sequence of steps performed. It also encouraged awareness of others and group connections.
Stage 3: Introduction to salsa and cha-cha. This stage aimed to increase muscle demand and required greater coordination between upper limbs and lower limbs. It was assumed that the greater energy required could stimulate an improvement in aerobic capacity. It also aimed to support release of physical tensions and associated emotions.
Stage 4: Consolidation and repetition of the already known dancing steps and choreographies. Participants were then encouraged to learn the basic steps of more complex dances such as rumba and tango that involved further core body engagement. By doing this, acknowledging and working though deeper emotional material became possible.
Stage 5: Participants were encouraged to choreograph their own dance, combining steps learned in previous months. At this point, participants were expected to not need the mirrors for instruction and to be confident to dance without the facilitators offering demonstrations. Psychologically it was the time when participants were encouraged to integrate movements and steps that were important for them and share their choreography with another.

In each country the programme was delivered by three facilitators per group of 10–18 women. Facilitators included experienced professional dancers with at least 5 years of experience (in the UK they also had additional dance movement therapy qualifications) and exercise professionals with qualifications in sports and movement sciences, reflecting the skills needed to deliver the core protocol. All facilitators attended 2 training days to ensure standardization of the protocol and its delivery, and to make it transferable and replicable in other contexts and countries. It was expected that different dance practitioners with diverse backgrounds and qualifications would be able to deliver this intervention. Depending on the qualifications of the facilitators and on participants' needs/preferences, stronger psychotherapeutic or dance emphasis was encouraged. It was expected that while standardization was encouraged, flexibility on qualifications allowed for variations on the delivery of the intervention to make it appropriate and relevant to the diverse organizational contexts, the different cultures of the participating countries and ultimately the individual needs of the participating women.

### Outcome Measures

Primary outcome measures included: anthropometric data (weight, waist, hip, arm, wrist circumferences), cardiorespiratory fitness (6 min walking test), several functional capacities (i.e., handgrip test, 30 s sit-to-stand, back scratch test, and Fullerton advanced balance scale). Quality of Life was also evaluated (cancer-related EORTC-QLQ C30 questionnaire) and constituted a secondary outcome. All the measures were taken before and after the intervention by trained researchers across the sites.

#### Anthropometric Measurements

The participants' weight was recorded in kilograms. In addition, a range of circumference measurements were taken in centimeters from the following points: waist, hip, three points along both left and right arms (lateral tip of acromion, most distal point of acromion process, and the midpoint) and left and right wrist. The waist circumference is an accurate and simple index of abdominal adiposity and when used together with the hip circumference, the waist to hip ratio can be a useful measure of body fat distribution (Ross et al., 2008; Seimon et al., 2018). Whilst these measurements alone cannot determine a future pathological condition, it could be a predictor of future disease such as cardiovascular diseases or type II diabetes.

#### Cardiorespiratory Fitness

**6 min walking test**: This test developed by the American Thoracic Society (ATS) (2002) measures the endurance and residual functional capacity of patients and is generally recommended for diagnostic purposes, it is seen as an indication of global health and it is positively correlated with quality of life (Galiano-Castillo et al., 2016).

With regards to validity with a cancer population Schmidt et al. (2013) found that the distance walked correlated significantly (*p* < 0.001) with VO_2_peak, the maximum exercise capacity (*r* = 0.67) and perceived physical function on the EORTC QLQ-C30 quality of life (cancer) questionnaire and physical function subscale (*r* = 0.55). Reliability was *r* = 0.93 (95% CI: +0.86; +0.97; *p* < 0.001) with a coefficient of variation 3%.

In our study, the test was performed to ATS guidelines for the 6 min walking test [American Thoracic Society (ATS) Committee on Proficiency Standards for Clinical Pulmonary Function Laboratories, 2002]. The test is self-paced i.e., the participant chooses the intensity of effort, walks at their preferred speed, can perform stops and use support. The test field was flat, 10 meters were marked out and the participants were asked to walk back and forth to cover as much distance as possible within the 6 min. The total distance was calculated in meters and was used as the outcome by which to compare changes in performance capacity. At the end of the test, each participant was asked to score their perceived exertion using the Borg scale from 1 to 10, with 1 being “really easy” and 10 being “maximal effort.”

#### Muscle Strength

**Handgrip**: The Handgrip test is seen as the simplest method for assessment of muscle function in clinical practice (Roberts et al., 2011) and can be useful for the assessment of disease and/or rehabilitation progression, in particular the assessment of upper limb impairment, overall fitness and is seen to be strongly related to functional status (Reuter et al., 2011).

Measurements of grip strength taken with the Jamar dynamometer have evidence for good to excellent (*r* > 0.80) test–retest reproducibility and excellent (*r* = 0.98) interrater reliability (Roberts et al., 2011). Validity has also been tested in relation to distance walked during the 6 min walking test (Reuter et al., 2011) with a sample of healthy adults, where a significant correlation was found between “distance” recorded by the 6 min walking test and “initial max” handgrip score for both the dominant and non-dominant hands (*p* = 0.017 and *p* = 0.016, respectively), as well as “distance” and “final max” the final handgrip scores, for the dominant and non-dominant hands (*p* = 0.003 and *p* = 0.007, respectively).

A Jamar hand dynamometer reads force in both kilograms and pounds, with markings at intervals of 2 kg or 5 lb, allowing assessment to the nearest 1 kg or 2.5 lb. For this study measurements were recorded in kg. The patient performed the test in a standing position with a 90-degree flexed forearm. The assistant set the instrument and after holding the device, the participant was asked to squeeze their hand as hard as possible for a few seconds, being careful to squeeze only once for each measurement. For each subject, three contractions were recorded per side (left and right) to the nearest kilogram. If the difference in scores was within 3 kgs, the test was completed. If not, a fourth measurement was taken and the lowest value was crossed off. The mean value of the end three results was recorded as the final score with a higher handgrip score indicates higher functioning/strength.

**30 s sit-to-stand test**: This test can assess patient's muscular endurance (Millor et al., 2013), analyze functional lower extremity strength, transitional movements, balance, and fall risk (Bohannon et al., 2010) and is considered a predictor of physical capacity (Jones et al., 1999). With regards to validity and reliability, in a sample of elderly participants, the sit-to-stand test has proven to have excellent test–retest reliability (*r* = 0.89, 95% CI 0.79–0.93), interrater reliability (*r* = 0.95, 0.84–0.97), and criterion validity when compared to weight adjusted leg press performance (Jones et al., 1999).

The test is conducted with participants fully seated in the middle of a chair with a seat height of 17 inches, with a straight back and feet approximately a shoulder width apart and placed on the floor at an angle slightly back from the knees. One foot should be slightly in front of the other to help maintain balance. Arms are crossed at the wrists and held against the chest. The participant is encouraged to complete as many full stands as possible within 30 s, ensuring that they fully sit between each stand. The final score is the number of recorded stands in the 30 s period (Shirley Ryan Ability Lab, 2020) with a higher number of stands indicating higher functioning/muscle strength.

#### Balance

**Fullerton advanced balance scale**: This test involves a series of 10 tasks aimed to challenge the visual, proprioceptive, and vestibular systems (Evans et al., 2019). In a population of functionally independent older adults, the results of the Spearman rank correlation analysis indicated convergent validity produced a significant (*P* < 0.01) with test–retest reliability as r=0.96, and interrater reliability in the range of *r* = 0.91–0.95, when the test is administered by trained raters (Rose et al., 2006). Klein et al. (2011) also found the Fullerton advanced balance scale to have high person and item separation reliability, suggesting that the tool can discriminate among participants of varying balance abilities.

Participants are scored on how well they perform each of the 10 tasks including: standing with feet together and eyes closed, reaching forward with an outstretched arm to retrieve a pencil at shoulder height, completing a 360° turn in both the right and left directions, stepping onto and over a 6 inch high bench, a tandem walk, standing on one leg, standing on foam with eyes closed, performing a two-footed jump for distance, walking with head turns at an established pace, and responding to an unexpected trunk perturbation. Each task is scored on a scale of 0–4, and the scores for the 10 tasks are added together to form a composite score. Composite scores for the Fullerton advanced balance scale can range from 0 to 40, with higher scores suggesting an increased ability to maintain balance (Evans et al., 2019).

#### Flexibility

**Sit-and-reach test**: This test measures the flexibility of the back and hamstrings muscles and can be useful to evaluate the functional ability of legs in terms of walking speed and dynamic balance (Wells and Dillon, 1952; Jones et al., 1998). A meta-analysis of 34 sit-and-reach studies by Mayorga-Vega et al. (2014) showed that all sit-and-reach tests had a moderate mean criterion-related validity for estimating hamstring extensibility (rp = 0.46–0.67), but a low mean for estimating lumbar extensibility (rp = 0. 16–0.35). Reliability in a study with a sample of professional futsal players showed a high reliability (4.48% typical error; 0.84% change in the mean, 0.95 intraclass correlation coefficient (Ayala et al., 2012).

A step or box with a centimeter rod is required to perform the test. The participant sits on the ground with their legs extended forward and pressed to the floor. Feet should be in plantar flexion and placed flat against the box. With their arms forward and palms facing downwards, the participant performs a front flexion of the torso reaching forward along the measuring line as far as possible. The position should be held for 1–2 s while the distance is from their toes to their fingertips is recorded. If their fingers are past their toes, the results are positive, if the fingers are behind the toes, the results are negative. The measurements are in cm, with a higher score indicating higher flexibility.

**Back Scratch Test**: This is a test for shoulder flexibility and mobility and can also be utilized to evaluate the range of motion (ROM) of the shoulder joint that could be seriously compromised by surgery in breast cancer patients (Rikli and Jones, 1999; Jones and Rikli, 2002; Rózańska-Kirschke et al., 2006). A reliability study in 71 healthy older women (Dewhurst and Bampouras, 2014) showed high reliability (0.97–0.99 intraclass correlation coefficient; 0.92 cm typical error) of the back scratch test, replicating the high inter-day reliability of this test previously found by Rikli and Jones (1999).

This test is performed in a standing position. The upper arm performs a combined movement of flexion, external rotation and abduction; while the lower one is a combined movement of extension, internal rotation and adduction. The distance between (or overlap of) the tips of the middle fingers is measured with a centimeter tape, and one side is executed at a time. If the fingers do not touch it will result in a negative score, if they overlap, the result is a positive score. The score is recorded in centimeters, with a higher positive score meaning higher flexibility and mobility.

#### Quality of Life Measure

**EORTC QLQ-C30 Version 3 Cancer-related quality of life questionnaire:** The European Organisation for Research and Treatment of Cancer (EORTC) Quality of Life group developed the EORTC QLQ-C30 (Aaronson et al., 1993) one of the most widely used health-related quality of life questionnaires in cancer research. The EORTC QLQ-C30 is a validated, self-reported measure composed of both multi-item scales and single-item measures. These include five functional scales (physical, emotional, role, cognitive and social), three symptom scales (fatigue, nausea/vomiting, and pain), a global health status/quality of life scale, and six single symptom items (Dyspnoea, insomnia, appetite loss, constipation, diarrhea, and financial difficulties). In the version used for this study (version 3), the first 28 items are rated on a response scale of “not at all” (1), to “very much” (4). The scoring algorithm results in the responses to values on a scale of 0 to 100 (Fayers et al., 2001). For the functional scales, a higher score corresponds to better functioning, likewise a higher score on the global health scale indicates higher quality of life. For symptom scales, a higher score corresponds to a higher level of symptoms/problems. EORTC also developed an overall quality of life summary score—calculated from several of the function and symptom scales. In oncology research, this single summary score appears to be a meaningful, reliable, and robust measure, with a higher score indicating a higher quality of life (Kasper, 2020). A validation study of the domains of the core EORTC quality of life questionnaire found substantial construct validity (Niezgoda and Pater, 1993) with all inter-scale correlations statistically significant (*p* < 0.01) (Aaronson et al., 1993). In a study with 348 Kuwaiti women with breast cancer the QLQ30 internal consistency values for the full questionnaires and their multi-item scales (i.e., ≥3 items) met the 0.7 Cronbach's alpha value requirement for the responses of the patients (Alawadhi and Ohaeri, 2010).

### Data Analysis

The number of participants who completed both the pre and post intervention outcome measures were different across outcome measures. For this reason, a per protocol (PP) approach to data analysis was chosen.

The analysis was conducted using the Statistical Package for the Social Sciences (SPSS) version 25 for Windows (IBM, 2020). At first each variable was checked for normality using the Shapiro–Wilk test (Shapiro and Wilk, 1965). As a result of this process outliers were identified and cleaned leading to each variable having different numbers of participants (*n*). The variables with no normal distribution were tested for pre and post intervention differences using the non-parametric Wilcoxon signed ranked test. Median and interquartile range were chosen to represent statistical dispersion for these variables. For variables that showed normal distribution, parametric paired *t*-tests were performed, whilst mean and standard deviation scores were chosen as appropriate descriptors of measures of central tendency.

To fully understand the impact of the intervention Eta squared values which reflect on the amount of variance accounted for in the sample were also calculated. Those values were used to determine the effect size for all tests performed by converting them to Cohen's d using a free online software by Psychometrica (Lenhard and Lenhard, 2016). The suggestions offered by Cohen (1988) were used to interpret the magnitude of effect sizes (<0 = adverse effect; 0.2–0.4 = small effect; 0.5–0.7 = intermediate effect; 0.8–>1 = large effect).

## Results

Over the course of the 16 week programme, 12 women dropped out (Bulgaria = 5, UK = 4, Netherlands = 3). Reasons included illness, travel problems, difficulties attending during winter/weather, inability to commit, childcare issues. An additional four were excluded from the dataset as they did not attend a minimum of 50% of the sessions. The remaining 54 were deemed as having followed the programme (*N* = 54). Participants were distributed as follows: Netherlands *n* = 15, Italy *n* = 14, Lithuania *n* = 11, Bulgaria *n* = 8, UK *n* = 6. The mean age was 53.51 (Standard Deviation = 7.99). Table 1 shows treatments for the women who completed the programme before and during the intervention.

**Table 1:** Treatments Pre and During Intervention per % of women who completed the programme.

	**Treatment Pre Intervention**				
	**Chemotherapy**	**Radiotherapy**	**Surgery**	**Hormone therapy**	
% of participants	49	64	87	66	
	**Treatment During Intervention**^**a**^				
	**Chemotherapy**	**Radiotherapy**	**Surgery**	**Hormone therapy**	**Other cancer related medication**
% of participants	0	2	9	50	23

*Figures rounded to nearest %*.

a *Estimations only are provided for treatment during intervention (hormone therapy and other cancer related medication) as one country's dataset was unavailable. These figures are calculated using average percentages from other countries' datasets*.

Despite exclusion criteria involved no indication of metastasis or scheduled surgery/chemotherapy/radiation treatment for the duration of the intervention, in reality 2% of women (*n* = 1) underwent further radiotherapy and 9% (*n* = 5) had unscheduled cancer related surgery which included cosmetic surgery, bilateral oophorectomy (ovaries removal), uterine polyps and cholecystectomy (gallbladder removal).

The anthropometric and fitness scores pre and post intervention are shown in Table 2. Weight, waist, arm, forearm, wrist (right and left in all cases), 30 s sit-to-stand, Fullerton, sit-and-reach and back scratch for the left arm all had *p* < 0.05 on the Shapiro Wilk test. They were therefore, regarded as lacking normal distribution and were subjected to Wilcoxon's signed-ranked test. The remaining parameters were also tested for normality and reached a *p* > were regarded as normally distributed; paired *t*-tests were used to establish differences in these parameters.

**Table 2:** Anthropometric and Fitness Measures Pre–Post Intervention Comparisons.

	**Pre**	**Post**	**Sample size**	**Score**	**Significance**	**Effect size**	
	***Wilcoxon's signed-ranked test***					***Cohen's d***	
	***Mdn ± IQR***	***Mdn ± IQR***	***n***	***Z***	***P* (one-tailed)**	***η^2^***	***d***
Weight (kg)	69.84 ± 15.32	65.50 ± 15.45	38	−3.100	0.001^**^	0.25	1.16
Waist Circ. (cm)	86.00 ± 17.75	86.25 ± 19.63	52	−1.187	0.118	0.02	0.33
Arm R (cm)	29.00 ± 5.25	29.00 ± 6.00	46	−0.480	0.316	0.00	0.14
Arm L (cm)	29.00 ± 5.00	29.00 ± 5.00	35	−1.137	0.128	0.03	0.38
Forearm R (cm)	26.00 ± 2.05	25.75 ± 3.00	46	−2.541	0.006^*^	0.14	0.80
Forearm L (cm)	26.00 ± 3.00	26.00 ± 3.50	35	−1.669	0.048^*^	0.07	0.58
Wrist R (cm)	17.00 ± 2.00	17.00 ± 2.00	46	0.531	0.298	0.00	0.15
Wrist L (cm)	17.00 ± 1.00	16.50 ± 2.50	35	−0.406	0.342	0.00	0.13
Sit-to-Stand (no)	13.50 ± 3.50	15.00 ± 5.25	50	2.825	0.003^*^	0.15	0.86
Fullerton (pt)	37.00 ± 9.00	36.00 ± 8.00	50	1.247	0.106	0.03	0.35
Sit-and-Reach (cm)	1.00 ± 10.00	3.00 ± 11.50	51	2.033	0.021^*^	0.10	0.68
Back Scratch L (cm)	14.00 ± 21.75	13.00 ± 20.50	52	−0.088	0.465	0.00	0.02
	***Paired t-test***					***Cohen's d***	
	***M±SD***	***M±SD***	**n**	***t***	***P*** **(one-tailed)**	***η******^2^***	***d***
Hip Circ. (cm)	104.94 ± 8.66	103.77 ± 8.25	50	2.870	0.003^*^	0.14	0.81
Handgrip R (kg)	22.78 ± 5.74	24.70 ± 5.79	51	−3.564	0.001^**^	0.19	0.99
Handgrip L (kg)	20.76 ± 5.34	22.64 ± 5.67	50	−3.909	0.000^**^	0.23	1.10
Back Scratch R (cm)	10.68 ± 11.13	10.85 ± 11.38	52	−0.273	0.393	0.00	0.07
6 Min Walking (mt)	521.36 ± 71.28	557.60 ± 87.62	52	−5.078	0.000^**^	0.322	1.38

*Kg, kilograms; cm, centimeters; no, numbers; pt, score; R, right; L, left; Circ, circumference; mt, meters*.

*M, mean; Mdn, median; SD, Standard Deviation*.

*Higher scores indicate higher functioning on the handgrip, back scratch test, 30 s sit-to-stand, sit-and-reach, Fullerton advanced balance scale and 6 min walking test*.

** *accepted at p <0.001*;

* *accepted at p < 0.05*.

*Cohen's d: adverse effect = <0; no effect = 0.0–0.1; small effect = 0.2–0.4; intermediate effect = 0.5–0.7; large effect = 0.8–>1.0*.

As shown in Table 2, the use of Wilcoxon's signed ranked test showed significant differences for the following variables: weight (Z = −3.10, *p* =0.001, η2 = 0.25, Cohen's d = 1.16), right forearm circumference (Z = −2.54, *p* =0.006, η2 = 0.14, Cohen's d = 0.80) and left forearm circumference (Z = −1.67, *p* =0.048, η2 = 0.07, Cohen's d = 0.59); all indicating improvements. Cohen's d calculated showed that these improvements had intermediate to large effect sizes. The Wilcoxon's signed ranked test also revealed statistically significant differences with improved scores after the intervention in the 30 s sit-to-stand test (Z = 2.82, *p* = 0.003, η2 = 0.15, Cohen's d = 0.86) and sit-and-reach (Z = 2.03, *p* = 0.021, η2 = 0.10, Cohen's d = 0.68). The Cohen's d scores indicate intermediate to large effect sizes.

Furthermore, paired *t*-tests performed indicated some statistically significant changes, while the calculation of Cohen's d showed large effect sizes for hip circumference (t = 2.87, *p* = 0.003, η2 = 0.14, Cohen's d = 0.81), right handgrip strength (t = −3.56, *p* = 0.001, η2 = 0.19, Cohen's d = 0.99), left handgrip strength (t = −3.91, *p* = 0.000, η2 = 0.23, Cohen's d = 1.10) and the 6 min walking test (t = −5.08, *p* = 0.000, η2 = 0.32, Cohen's d = 1.38). While the hip circumference score was reduced after the intervention, handgrip for both right and left hand and the 6 min walking test showed an increase; in all cases the scores changed positively.

Table 3 shows scores on functional aspects, symptom scales and the summary score of the quality of life measure used, namely the EORTC QOL-C30.

**Table 3:** EORTC QLQ-C30 (V3) Cancer-Related Quality of Life Questionnaire Pre–post Intervention Comparisons.

	**Pre**	**Post**	**Sample size**	**Score**	**Significance**	**Effect size**	
	***Wilcoxon's signed-ranked test***					***Cohen's d***	
	***Mdn ± IQR***	***Mdn ± IQR***	***n***	***Z***	***P (one-tailed)***	***η****^2^*	***d***
**QLQ30 functional scale**							
Physical	80.00 ± 20.00	86.67 ± 20.0	53	1.544	0.062	0.04	0.43
Role	83.33 ± 33.33	83.33 ± 33.33	52	0.328	0.372	0.00	0.09
Emotional	75.00 ± 33.34	83.33 ± 33.33	51	2.822	0.003^*^	0.15	0.86
Cognitive	83.33 ± 33.33	83.33 ± 33.33	51	0.327	0.372	0.00	0.09
Social	83.33 ± 37.50	83.33 ± 33.33	54	2.824	0.003^*^	0.14	0.83
Global	33.33 ± 20.83	21.17 ± 18.74	54	−0.571	0.303	0.00	0.15
**QLQ30 symptom scale**							
Fatigue	33.33 ± 36.12	33.33 ± 40.28	54	−2.832	0.003^*^	0.14	0.83
Nausea	8.34 ± 66.67	0.00 ± 0.00	52	−3.678	0.000^**^	0.25	1.18
Pain	33.33 ± 41.67	16.67 ± 33.33	53	−1.409	0.080	0.03	0.39
Dyspnoea	0.00 ± 33.33	0.00 ± 33.33	54	−0.265	0.396	0.00	0.07
Insomnia	33.33 ± 66.67	33.33 ± 33.33	49	−1.772	0.038^*^	0.06	0.52
Appetite loss	0.00 ± 33.33	0.00 ± 33.33	54	−1.134	0.129	0.02	0.31
Constipation	0.00 ± 33.33	0.00 ± 33.33	52	−0.504	0.307	0.00	0.13
Diarrhea	0.00 ± 0.00	0.00 ± 0.00	54	1.155	0.124	0.02	0.31
Financial	33.33 ± 66.67	33.33 ± 33.33	52	−1.863	0.031^*^	0.06	0.53
**QLQ30 summary score**							
Summary score	79.06 ± 17.13	83.89 ± 17.05	54	3.119	0.001^**^	0.17	0.93

*Scores are represented on a scale of 0 to 100. For the functional scales, a higher score corresponds to better functioning. Likewise, a higher score on the summary score indicates higher quality of life. For the symptom scales, a lower score corresponds to a reduction of problems*.

** *accepted at p < 0.001*;

* *accepted at p < 0.05*.

*Cohen's d: adverse effect = <0; no effect = 0.0–0.1; small effect = 0.2–0.4; intermediate effect = 0.5–0.7; large effect = 0.8–>1.0*.

When we tested for statistical significant changes and for effect sizes on this outcome measure, we found the following important results: there were statistically significant changes with a large effect on the emotional (Z = 2.82, *p* = 0.003, η^2^ = 0.15, Cohen's d = 0.86) and social functioning scales (Z = 2.82, *p* = 0.003, η^2^ = 0.14, Cohen's d = 0.83). On the scales relating to symptoms, there were statistically significant changes with intermediate to large effects for fatigue (Z = −2.83, *p* = 0.003, η^2^ = 0.14, Cohen's d = 0.83), nausea (Z = −3.68, *p* = 0.000, η^2^ = 0.25, Cohen's d = 1.18), insomnia (Z = −1.77, *p* = 0.038, η^2^ = 0.06, Cohen's d = 0.52) and financial difficulties (Z = −1.86, *p* = 0.031, η^2^ = 0.06, Cohen's d = 0.53). The total summary score (Z = 3.12, *p* = 0.001, η2 = 0.17, Cohen's d = 0.93) also showed a statistically significant change with a large effect size.

## Discussion

This pilot study aimed to measure the impact of the therapeutic dance programme titled Dancing with Health delivered across five European countries. Results suggest that study participants demonstrated positive changes in both physical as well as quality of life measure after attending at least 50% of the sessions offered in the Dancing with Health programme, despite the fact that over 10% had unplanned surgery and radiotherapy during their participation in the programme. The size of the intervention effects on the physical and psychosocial measures used in this study as measured through Cohen's d are also promising with a range of intermediate (>0.5) to large impact (>0.8).

Specific results are also worth discussing. For example, one important anthropometric finding involved *weight loss*. Obesity is a risk factor for breast cancer (Seiler et al., 2018) and weight gain is certainly a concern amongst women post-chemo and endocrine therapy (Vance et al., 2011). However, while there is a growing number of studies on the value of physical activity toward weight loss for cancer patients (Hardefeldt et al., 2018), the research literature is particularly thin on the use of dance for weight loss in the same population (Boing et al., 2017). Loo et al. (2019) is the only study with relevant results reporting decreased levels of circulating cytokines that are linked with obesity and inflammation. In this respect, findings from this study on the impact of the dance programme on weight loss are therefore, particularly important.

Weight loss may also explain the statistically significant reduction on *hip circumference* scores. However, similar changes on measurements on waist circumference were not found. It is possible that estrogen blockers such as tamoxifen, commonly used in hormone therapy [National Institute for Health and Care Excellency (NICE), 2018], may be responsible for lack of changes on this measure; half of the participants in this study were on hormone therapy during the intervention.

Interestingly, changes on *forearm* measurements were found, but no changes on circumference of arms and wrists. The use of gentle free movement in the sessions with no weight-bearing activities may be responsible for these results. The process of opening arms and reaching out to others during the different dances may have had an impact on the circumference of the forearms but not on any other parts of the arms. It is possible that participants, not being encouraged to use weight-bearing activities, have had no opportunities to reduce inflammation and edema on the arms and wrists, areas of change reported on other similar studies (Boing et al., 2017). It is also possible that further engagement of the upper body during the intervention could have had positive results on more measures. In this case, safety would need to be carefully considered given that breast cancer surgery particularly affects arms and shoulders.

Nevertheless, changes in *handgrip strength* for both right and left hand were statistically significant. Participants appeared to be able to “get hold of their lives” with both hands. This was a particularly promising result since strong handgrip is a positive prognostic indicator for reduced mortality and overall health post hospitalization and surgery (Bohannon, 2008). Such findings echo work by Kaltsatou et al. (2011) on Greek group dance reporting similar positive changes.

Positive changes were also detected in the *30 s sit-to-stand* exercise: participants in the dance programme developed lower limb strength as also indicated in other dance studies (Bohannon et al., 2010). They appeared to be able to “get on their feet” and “get back up,” and thus they may had become more able to manage the health problems they were faced with. This test is widely used and validated in aging populations (Macfarlane et al., 2006; Rikli and Jones, 2013; McAllister and Palombaro, 2020) and has been used with cancer patients with head and neck concerns (Capozzi et al., 2015) as well as within a wider cancer rehabilitation exercise programme (Smith et al., 2016). Results from this study supports its use in future research suggesting it is a sensitive enough method to pick changes in strength following a dance interventions.

Similar assumptions can be held regarding flexibility. Although there were no significant changes on the back scratch test, the positive results for the *sit-and-reach test* suggests that the overall flexibility of the participants was improved. Participants appeared to be able to “reach further” in their lives, a potential metaphor of better coping with their illness. Relevant research results for breast cancer patients suggest that increased flexibility is linked with improvements on several health measures including quality of life (Kolden et al., 2002). It is possible that the improved flexibility found in this study is linked with improvements in quality of life, an association that deserves further explored in future studies.

Finally, the cardiorespiratory fitness of the participants in the study also improved as indicated by the *6 min walking test*. This is particularly important given that the dance intervention was offered for only 2 h per week, 1 h less than the recommended weekly input suggested by Boing et al. (2017). Still, there were clear cardiorespiratory benefits for the participants. A large total number of sessions offered in this study (larger than the recommended dosage by Boing et al., 2017) within the recommended moderate intensity might be responsible for this positive outcome. Furthermore, it is possible that, as suggested in the literature (Schmidt et al., 2013; Galiano-Castillo et al., 2016), improvements in cardiorespiratory fitness as recorded by the 6 min walking test can be linked with other health indicators such as quality of life. Further exploration of this relationship is needed within the context of dance research.

Finally, positive changes were found on perceived *quality of life*. Positive changes in the total score for the quality of life measure, as well as large improvements on the emotional and social scales, do back similar findings in the literature. For example, both the systematic review by Boing et al. (2017) and the Cochrane Review by Bradt et al. (2015) highlight that dance and dance movement therapy programmes can make an important contribution to the quality of life of women recovering from cancer. Qualitative findings (Dibbell-Hope, 2000; Serlin et al., 2000, 2017; Ho et al., 2016) explore this issue further by referring to working through the body (using the body as a vehicle for emotional exploration), as an opportunity to create new associations and perspectives of/with themselves, as well as share emotional difficulties within a supportive environment. Dance movement therapy literature in particular highlights the need to do this in a safe way, stressing the importance of the presence of a qualified dance movement therapist as a facilitator in such groups. In the absence of this, the need for training into safe uses of dance with this client group and awareness of limits of one's practice seem to be essential pre-requisites.

*Cancer-related fatigue* was also part of the cancer-related quality of life measure (see symptom scales on Table 3). Given how prevalent fatigue is amongst women with cancer (Schneider et al., 2003), identifying perceived improvement on this measure amongst the participants in this study was important and in accordance with relevant literature (Sturm et al., 2014; Boing et al., 2017; Loo et al., 2019). Lower scores in perceived fatigue may also be linked with improved physical fitness. Arnett et al. (2008) argue that when cardiorespiratory fitness is improved, greater aerobic reserve may be associated with a delayed onset of muscle fatigue, improvements in functionality and physical independence. Fatigue may therefore, explain why people avoid engaging in physical activity (Rethorst et al., 2018) but at the same time the need to reduce fatigue is why physical activity can be helpful post-surgery, acting as an important antidote. Making physical activity attractive and safe through creative work can be one of the ways of motivating participation; people may engage more readily with something they find pleasant. This may also have a direct impact on reducing nausea and improving sleep as we found for the participants in this study. Although physical activity literature reports on such changes (e.g., Kreutz et al., 2019), until now, there have been no reports that dance and/or dance movement therapy may be linked with improved sleep and reduction in nausea for women recovering from breast cancer.

Interestingly, unlike other studies on dance for Parkinson's disease (Hackney and Earhart, 2010; Duncan and Earhart, 2012) for example, we found no changes in balance measures. It is possible that people with Parkinson, participants in this study did not present balance issues before the intervention, creating a ceiling effect on the pre-intervention measures. Therefore, improvements on pre-existing good scores on balance were not possible.

The study was ambitious, collecting data from several countries and thus with several inconsistencies in the data collection process leading to an inevitable heterogeneity. Different recruitment processes and settings could explain differences in the samples across the countries. In the UK, Lithuania, and Bulgaria for example, the study took place in the community in cultural organizations and sports centers, settings that were very different from the medical center in the Netherlands, and the university in Italy. Participants fell within the specifications of the inclusion/exclusion criteria, but different countries did not collect demographic information in a consistent manner. Furthermore, the facilitators had quite different backgrounds, qualifications, and levels/types of experiences. In the UK for example, the facilitators were all qualified as dance movement therapists in addition to having expertise in Latin dance teaching and exercise. These differences must have had an inevitable impact on the way the intervention was delivered despite the rigorous training of the facilitators prior to the commencement of the work. These differences sat on top of cultural and practical variations. In the UK for example, the intervention was delivered once a week because both the facilitators and the participants, coming from further afar could not commit to being in the particular setting twice a week. Longer sessions were offered instead. As exercise frequency is a very important variable for physical fitness, it is possible that improvements on physical measurements have been affect by other factors (e.g., overall increase in daily physical activity levels), rather than the dance intervention solely. Other variations from the protocol were not reported. Adherence measures were not used to establish numerical diversion from the agreed protocol. Nevertheless, despite the reported differences, the study was possible and the intervention impactful, alleviating concerns around the feasibility of the study across five countries. Furthermore, the heterogeneity of the group reflected common practice, indicating how this intervention could be implemented after the completion of the study without losing important characteristics.

Further limitations of the study design were in the absence of a control group and randomization which restricted the study's ability to conclude a causal association between the Dancing with Health intervention and the outcome measures with certainty. The absence of control group presented difficulty in measuring and controlling for important confounding variables, particularly unmeasured confounding variables such as ongoing medications, age, lifestyle, social-cultural background. The presence of an active control such as an exercise group could have allowed for comparisons of the effects of dancing with other types of traditional types of exercise already studied with breast cancer patients (e.g., aerobic and resistance training) (Herrero et al., 2006). This type of active control would give a more real magnitude of the fitness gains provided by regular dance practice and can be considered for future studies. In the current study however, due to the absence of a control group there is a possibility for wrongly concluding that the positive effects are due to the intervention when in reality they are due to chance leading to the possibility of committing a Type II error. Furthermore, the presence of several outliers in the dataset leads to a threat from regression to the mean (Morton and Torgerson, 2003; Bland and Altman, 2008). For these reasons, causation cannot be assumed; findings need to be interpreted with caution and cannot be generalized.

Further caution is needed due to the use of PP analysis. It has been argued that the PP approach can potentially offer an estimate of the true efficacy of an intervention among those who completed the intervention programme as planned (Ranganathan et al., 2016), who, in this study, were all those who attended at least 50% of the sessions. Nevertheless, since the PP approach considers only those who attended the intervention it increases the risk for distorted representation of the real-life situation as it is likely to show an exaggerated intervention effect (Ranganathan et al., 2016). Reporting both Intention to treat (ITT) and PP analysis has been argued as an option to address the issue of overestimation using imputation techniques by Schulz et al. (2010) in the CONSORT statement. However, the quasi-experimental design of this study restricted the implementation of an ITT approach and hence there is an expected bias influencing the estimate of true efficacy of the intervention, limiting the results to those who attended the intervention.

We would also suggest that the type of methods included (or not included) in the study needs additional consideration. For example, nutritional habits were not monitored during the intervention. Any change on weight, hip or forearm could be due to changes in nutritional habits. Absence of data on this domain limits our capacity to attribute changes to the programme only. An overall measure of physical fitness is also missing. Although it was planned initially for the International Physical Activity Questionnaire (IPAQ) to be included in this study, different versions were used across countries, which prevented the research team from creating a coherent data set. Furthermore, data from the EORTC QLQ-FA12 Cancer-related fatigue was collected across countries, but the information included in this questionnaire was also captured in the quality of life EORTC QLQ-C30 measure used under the symptom measures. For this reason, we decided to not include the results from this measure in this paper. Instead, and as a way of strengthening our understanding of the psychological impact of the intervention, in both the Netherlands and the UK additional measures for depression and anxiety were used, which were perceived as balancing out the more physical data collected. However, since these measures were used in only two countries, they did not constitute complete data sets for analysis and reporting purposes. Finally, in the UK study, it seemed important that qualitative data were collected alongside movement-based analysis of segments from the video recorded sessions. It was decided that these additional data sets could offer information on the process of implementing the programme and expand our understanding of some of the quantitative outcome-based results. Again, this type of data was collected in one site/country only and can be considered for inclusion in future studies. Further qualitative information about the intervention could be gathered across sites, informed by movement-based observations used in dance movement therapy. Assessing the movement quality of session participants before, during, and after sessions offers additional, insightful and potentially useful qualitative data not previously considered in the context of complex interventions. Furthermore, video recording dance sessions can lead to an easy reproduction of the programme. It can be used as a tool for quantitative analysis of data related to exercise tolerance and prescription. For example, it is possible to select the types of moves at moderate-vigorous intensity, the number of repetitions of selected dance routines, effective exercise time (ways in which dance routines are performed, with or without pauses) and so on. The use of video as a method of data collection can therefore, also be valuable in future studies.

As a preliminary study attempting to offer a first evaluation of the intervention, it axiomatically requires further refinements of processes and intervention design, as well as even though improvement in the design, the choice of measures and the analysis of the results. However, this remains an extensive evaluation, which, as with all complex interventions, is perceived as an important first step before larger studies are completed [Medical Research Council (MRC), 2019]. Research informing the design, type or optimal duration of such interventions is useful before a sufficiently powered study is conducted. In future studies, the presence of a control group is certainly recommended that can allow for the calculation of the sample size.

Finally, although a 3 months follow up was included in the current study, the impact of Covid-19 did not allow for all the countries to complete follow up measures. In further studies, follow up measures are certainly important to consider and report on. Future studies could also include a longer follow-up period to better elucidate potential benefits and sustainability of the intervention presented here.

## Conclusions

Results from this study shows that the Dancing with Health programme had both physical benefits on anthropometric measures and fitness levels, as well as psychosocial benefits for women with breast cancer. We found positive changes on weight, hip and forearms as well as on changes on cardiorespiratory fitness, overall flexibility and strength. The quality of life measure also indicated positive changes after the intervention.

Furthermore, the study highlights that joint effort across different countries can enable the development of an intervention which, with cultural variations, can be compared and treated as sufficiently homogeneous. Whilst most breast cancer dance studies are small, and larger randomized control trials are needed that are sufficiently powered, together our findings indicate that dance, or an integrated dance and exercise programme, could be a viable way of encouraging breast cancer patients to participate in physical activities. This attractive intervention for women can be engaging, having the potential to benefit them physically as well as improve the quality of their lives at a time when additional and holistic support is certainly needed.

## Data Availability Statement

The original contributions generated for the study are included in the article/supplementary material, further inquiries can be directed to the corresponding author/s.

## Ethics Statement

The studies involving human participants were reviewed and approved by University Medical Centre Utrecht, Netherlands and the Research Ethics Committee of the Faculty of Health and Social Care at Edge Hill University. The patients/participants provided their written informed consent to participate in this study.

## Author Contributions

VK was the project lead for the UK and led underpinning research and the writing of the paper. JS was responsible for the day to day running of the project and involved in the writing of the paper along with ID-S and JO-T. APars and SA did the statistical analysis. HV and RvdB had overall responsibility for the project in the Netherlands. HV contributed to the paper. YD, SD, IZ, and ID have been involved in the preparation, implementation, and data collection for the project in Bulgaria. AM was responsible for the project in the Lithuania. AB and AA were involved in the recruitment of participants in Italy. DF and AF offered support in the management of the project. SM and APari had responsibility for the project in Italy, while EG and CC had responsibility for the day to day running of the project in the same country. EG also contributed to the writing of the project, while ET supported the data collection. All authors contributed to the article and approved the submitted version.

## Conflict of Interest

The authors declare that the research was conducted in the absence of any commercial or financial relationships that could be construed as a potential conflict of interest.
